# Effect of Yoga Therapy on Insomnia Severity and Systolic Blood Pressure in Aged Women: A 12-Week Intervention Study Conducted in Kerala

**DOI:** 10.7759/cureus.57169

**Published:** 2024-03-29

**Authors:** Deepak Mathew, Muthulakshmi Rangasamy

**Affiliations:** 1 Yoga Sciences and Therapy, Meenakshi Academy of Higher Education & Research (MAHER), Chennai, IND; 2 Physiology, Meenakshi Medical College Hospital & Research Institute (MMCHRI), Chennai, IND

**Keywords:** stress, aged women, systolic blood pressure, insomnia, yoga

## Abstract

This study aimed to investigate the effects of a 12-week yoga therapy intervention on insomnia severity and systolic blood pressure in older women experiencing insomnia. A sample of 30 participants, aged between 60 and 70 years, underwent interventions, starting with an initial full-day workshop followed by 60-minute sessions held six days per week in the mornings. Women screening positive for insomnia using the Insomnia Severity Index Questionnaire were eligible for participation. The majority of participants were aged between 60 and 65 years, with a slight majority residing with a spouse. Economically, the group exhibited a diverse distribution, with a significant proportion classified as middle class. Educationally, the group also displayed a varied profile, with a range of educational qualifications. Pretests and post-tests were conducted to assess outcomes. The results revealed a significant reduction in Insomnia Severity Scores, with a mean decrease of 5.17 (±1.05), indicating a substantial effect size, from an initial average of 23.03 (±2.47) to 17.87 (±2.61) after the intervention. Similarly, there was a significant decrease in systolic blood pressure, with scores declining by a mean of 9.67 (±6.87), demonstrating a considerable effect size, from an average of 140.33 (±8.65) to 130.67 (±7.52). These findings suggest that yoga therapy holds promise as a non-pharmacological approach for alleviating insomnia symptoms and reducing systolic blood pressure in older women experiencing insomnia.

## Introduction

Insomnia stands as a common sleep disturbance marked by challenges in both falling asleep and staying asleep, resulting in daytime dysfunction and emotional distress [1]. It affects individuals of all ages but becomes increasingly common with advancing age, particularly among women [2]. Aged women, in particular, experience unique challenges related to sleep disturbances due to hormonal changes, comorbidities, and psychosocial factors [3]. Insomnia within this specific group is linked to a range of negative health consequences, such as heightened susceptibility to conditions like hypertension, cardiovascular diseases, cognitive deterioration, and a general decline in quality of life [4,5].

Despite the availability of pharmacological treatments, concerns regarding their long-term efficacy and potential adverse effects have led to a growing interest in non-pharmacological interventions for managing insomnia in aged women [6]. One such intervention showing promise is yoga therapy. Yoga, stemming from ancient roots in India, integrates bodily poses, controlled breathing techniques, and meditation to foster overall physical, mental, and emotional health [7]. Yoga therapy, which adapts yoga practices to address specific health conditions, has gained popularity as a complementary approach to conventional treatment modalities for various health issues, including insomnia [8].

Prior studies have shown the effectiveness of yoga therapy in enhancing sleep quality and alleviating symptoms of insomnia among diverse groups [9,10]. However, studies focusing specifically on aged women with insomnia are limited, warranting further investigation into the potential benefits of yoga therapy in this demographic [11]. Furthermore, considering the firmly established connection between insomnia and hypertension among elderly individuals [12], exploring the effects of yoga therapy on both insomnia severity and systolic blood pressure (SBP) could provide valuable insights into its holistic therapeutic potential.

Hence, the objective of this study is to explore the impact of a 12-week yoga therapy regimen on the severity of insomnia and SBP among older women experiencing insomnia. By examining the impact of yoga therapy on both sleep-related and cardiovascular outcomes, this research seeks to contribute to the development of effective, non-pharmacological interventions for improving the health and well-being of aged women experiencing insomnia.

## Materials and methods

Participants

The study recruited 30 aged women (age range: 60-70 years) experiencing insomnia symptoms. Participants were selected from local community centers and senior citizen groups.

Inclusion criteria

Basic physical fitness was required to ensure safe engagement in moderate yoga exercises. To minimize bias, individuals with minimal to no prior yoga experience were included. Additionally, women screening positive for insomnia using the Insomnia Severity Index Questionnaire were eligible for participation.

Intervention

The yoga intervention began with an initial full-day workshop, followed by 60-minute sessions held six days per week in the mornings over a 12-week period. The yoga therapy session comprised various components, including breathing awareness, loosening exercises, a series of asanas, relaxation techniques, breathing stretches, pranayamas, kriya, mantra chanting, and yoga nidra. These practices were designed to promote relaxation, flexibility, and mindfulness. The session began with breathing awareness, followed by loosening exercises and a series of asanas targeting different muscle groups. Relaxation techniques and breathing stretches were incorporated to further enhance relaxation and release tension. Pranayamas focused on controlled breathing, while kriya and mantra chanting aimed to purify the mind and enhance concentration. The session concluded with yoga nidra, a guided relaxation technique. Each component contributed to the holistic well-being of participants, addressing physical, mental, and emotional aspects. This structured approach ensured consistent engagement and progression throughout the intervention period. The specifics of yoga interventions are outlined in Table 1.

**Table 1:** Yoga module for Insomnia

Sl. no.	Name of yoga practice	Duration
1	Breathing Awareness	2 Minutes
2	Loosening Exercises: Pawanmuktasana Series1(BSY)	8 Minutes
3	Asanas: Bandha Hasta Utthanasana; Akarna Danurasana; Triyaka Tadasana; Kati Chakrasana; Marjarasana; Shashangasana; Bhujangasana; Suptha Badhakonasana; Ananda Balasana; Padottanasana with chair support; Pawanamukthasana; Suptha Vakrasana	12 Minutes
4	Relaxation	5 Minutes
5	Breathing Stretches: Hand Stretch Chest Expanding Breath; Abdominal Breathing in Supine; Candle Blowing	3 Minutes
6	Pranayamas: Bastrika; Nadi Shudi; Chandra Bedhana; Bramari	8 Minutes
7	Kriya: Jyoti Trataka	5 Minutes
8	Mantra: Aaa…Uuu..Mmm	5 Minutes
9	Yoga Nidra	10 Minutes
10	Breathing Awareness	2 Minutes
-	Total Duration	60 Minutes

The yoga therapy program was conducted by an experienced and certified yoga instructor with over 12 years of expertise in delivering yoga therapy to diverse populations. With a background in MSc. Yoga and currently pursuing a Ph.D. in Yoga, the instructor was well-equipped to tailor sessions addressing insomnia symptoms and hypertension in aged women.

Participants were encouraged and motivated to adhere to the yoga classes throughout the intervention period. Strategies such as regular reminders, personalized encouragement, and fostering a supportive group environment were employed to promote consistent attendance and engagement with the program.

Notably, there were no dropouts observed throughout the 12-week intervention period. Participants remained consistently engaged and committed to attending the yoga classes, demonstrating a high level of retention and adherence to the program.

Measurements

Insomnia severity was assessed utilizing the Insomnia Severity Index (ISI), a self-reported questionnaire specifically designed to measure the nature, intensity, and repercussions of insomnia symptoms [13]. SBP was measured using a sphygmomanometer. Demographic variables like Age, Spouse Status, Economic Status and Educational status were gathered using assessment form.

Procedure

Pretests for insomnia severity and SBP were conducted at baseline before the initiation of the yoga therapy intervention. Post-tests were administered at the end of the 12-week intervention period to assess changes in insomnia severity and SBP.

Data analysis

Descriptive statistics were employed to summarize the demographic features of the participants. Paired t-tests were utilized to examine the variations between pretest and post-test scores in terms of insomnia severity and SBP. The analysis included examining the association between effective values of SBP and selected demographic variables, as well as investigating the association between effective values of the Insomnia Severity Index (ISI) and selected demographic variables. These analyses aimed to elucidate any relationships between SBP or ISI values and demographic factors, contributing to a deeper understanding of how demographic characteristics might influence SBP and Insomnia Severity Index (ISI) levels within the study population.

## Results

Among the 30 elderly women experiencing insomnia, the majority, accounting for 20 (66.7%), were aged between 60 and 65 years, with 10 (33.3%) falling into the age range of 66 to 70 years. Regarding their living arrangements, a slight majority of 16 (53.3%) resided with a spouse, whereas 14 (46.7%) lived without one. Economically, the group exhibited a diverse distribution, with 10 (33.3%) categorized as low class, 18 (60%) as middle class, and a smaller two (6.7%) classified as high class. Educationally, the group also displayed a varied profile, with six (20%) having education below SSLC, 11 (36.7%) having attained SSLC education, seven (23.3%) possessing pre-degree qualifications, four (13.3%) holding undergraduate degrees, and two (6.7%) having obtained postgraduate qualifications. The above percentages are presented in Table 2.

**Table 2:** Frequency distribution of demographic variables SSLC, Secondary School Leaving Certificate; UG, Undergraduate; PG, Postgraduate

Demographic variables	Number of participants	Percentage of participants
Age in years ≤ 65	20	66.7
Age in years > 65	10	33.3
Presently living with spouse (Yes)	16	53.3
Presently living with spouse (No)	14	46.7
Economic status (Low)	10	33.3
Economic status (Middle)	18	60.0
Economic status (High)	2	6.7
Educational status (Below SSLC)	6	20.0
Educational status (SSLC)	11	36.7
Educational status (Pre degree)	7	23.3
Educational status (UG)	4	13.3
Educational status (PG)	2	6.7

**Figure 1:**
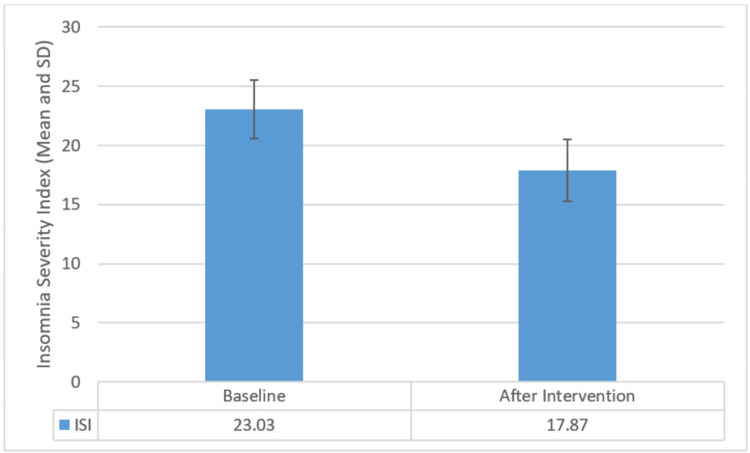
Bar graph showing comparison of means of Insomnia Severity Index scores before and after intervention with error bars showing standard deviation. SD, standard deviation

**Figure 2:**
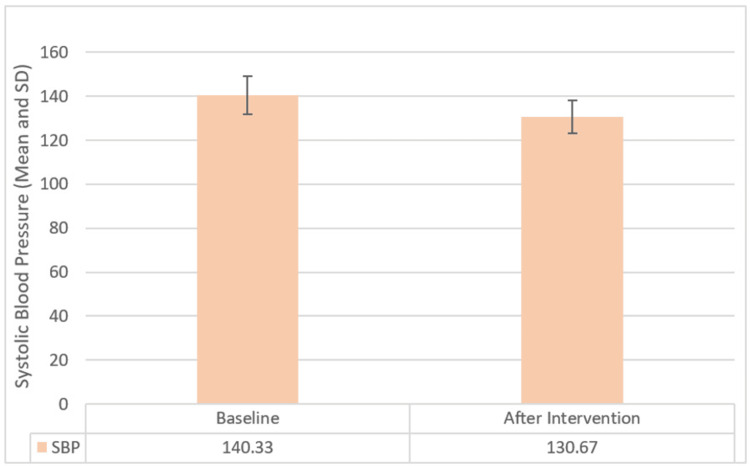
Bar graph showing comparison of means of systolic blood pressure scores before and after intervention with error bars showing standard deviation SD, standard deviation

The results of the study showed a notable reduction in Insomnia Severity Scores, with a mean decrease of 5.17 (±1.05), indicating a substantial effect size, from an initial average of 23.03 (±2.47) to 17.87 (±2.61) after the 12-week yoga intervention. This reduction was statistically significant, as evidenced by a calculated paired test value of t=26.870, surpassing the tabulated value of p<0.001. Similarly, there was a significant decrease in SBP, with scores declining by a mean of 9.67 (±6.87), demonstrating a considerable effect size, from an average of 140.33 (±8.65) to 130.67 (±7.52). The statistical significance of this decrease was supported by a paired test value of t=7.707, exceeding the tabulated value of p<0.001. Findings are illustrated in Figures 1, 2.

No statistically significant association was observed between the effective value on SBP and the selected demographic variables. Details are in Table 3.

**Table 3:** Association between effective value on systolic blood pressure and selected demographic variables n, number of participants; SD, standard deviation; SSLC, Secondary School Leaving Certificate; UG, Undergraduate; PG, Postgraduate; P-value of <0.05 is considered significant

Demographic variables	Systolic Blood Pressure n = 30			
	n	Mean	SD	F and t test value and p value
Age in years ≤ 65	20	10.20	6.99	t = 0.595 p = 0.557 (N.S)
Age in years > 65	10	8.60	6.87	
Presently living with spouse (Yes)	16	7.75	8.35	t = 1.685 p = 0.103 (N.S)
Presently living with spouse (No)	14	11.86	3.88	
Economic status (Low)	10	8.60	6.67	F = 0.709 p = 0.501 (N.S)
Economic status (Middle)	18	9.67	7.27	
Economic status (High)	2	15.00	1.41	
Educational status (Below SSLC)	6	8.00	8.39	F= 1.294 p = 0.299 (N.S)
Educational status (SSLC)	11	11.64	5.05	
Educational status (Pre degree)	7	11.71	3.55	
Educational status (UG)	4	7.00	7.39	
Educational status (PG)	2	2.00	16.97	

No statistically significant association was found between the effective value on Insomnia Severity Index (ISI) and the selected demographic variables, except for age group. Details are in Table 4.

**Table 4:** Association between effective value on Insomnia Severity Index and selected demographic variables n, number of participants; SD, standard deviation; SSLC, Secondary School Leaving Certificate; UG, Undergraduate; PG, Postgraduate; P-value of <0.05 is considered significant

Demographic variables	Insomnia Severity Index n = 30			
	n	Mean	SD	F and t test value and p value
Age in years ≤ 65	20	5.50	0.89	t = 2.706 p = 0.011 **
Age in years > 65	10	4.50	1.08	
Presently living with spouse (Yes)	16	5.44	0.89	t = 1.541 p = 0.135 (N.S)
Presently living with spouse (No)	14	4.86	1.17	
Economic status (Low)	10	5.30	0.82	F = 0.463 p = 0.634 (N.S)
Economic status (Middle)	18	5.17	1.20	
Economic status (High)	2	4.50	0.71	
Educational status (Below SSLC)	6	5.17	1.17	F= 0.406 p = 0.803 (N.S)
Educational status (SSLC)	11	4.91	1.30	
Educational status (Pre degree)	7	5.57	0.53	
Educational status (UG)	4	5.25	0.96	
Educational status (PG)	2	5.00	1.41	

## Discussion

The results of this study offer valuable insights into the potential advantages of utilizing yoga therapy as a non-pharmacological approach for addressing insomnia severity and SBP in older women. The significant reductions observed in both insomnia severity and SBP post-intervention highlight the holistic therapeutic potential of yoga in addressing sleep-related and cardiovascular health outcomes in this demographic.

The efficacy of yoga therapy in improving sleep quality and reducing insomnia symptoms aligns with previous research demonstrating its effectiveness across various populations [9,10]. Research has demonstrated that yoga practices, encompassing physical poses, breathing techniques, and meditation, can influence physiological mechanisms related to sleep regulation, such as the autonomic nervous system and the hypothalamic-pituitary-adrenal axis. This fosters relaxation and helps in reducing stress [8]. Moreover, the mindfulness-based components of yoga may enhance self-awareness and acceptance, leading to decreased pre-sleep arousal and rumination, common contributors to insomnia [8].

The decreases observed in SBP after the yoga therapy intervention align with prior research emphasizing the potential blood pressure-lowering effects of yoga [14,15]. Studies have demonstrated that yoga practices, including pranayama (breathing exercises) and meditation, can trigger relaxation responses, decrease activity in the sympathetic nervous system, and enhance endothelial function. These factors collectively contribute to the reduction of blood pressure levels [8,14]. Given the strong association between insomnia and hypertension in older adults [4], the simultaneous improvement in both measures points to yoga therapy's beneficial effects on older women's sleep and cardiovascular health.

While the findings of this study are promising, several limitations warrant consideration. The lack of a control group limits the ability to infer causality and control for potential confounding variables. Further investigation utilizing randomized controlled trials with increased sample sizes and extended follow-up durations is necessary to validate the effectiveness and durability of the observed effects. Additionally, factors such as adherence to the yoga intervention, participant characteristics (e.g., comorbidities), and the specific components of the yoga program may influence outcomes and need to be investigated extensively in future research.

## Conclusions

In conclusion, this study contributes valuable insights into the potential benefits of yoga therapy for addressing insomnia severity and reducing SBP among elderly women. Moving forward, there are opportunities to enhance the study's methodology to further strengthen its validity and generalizability. Future research could consider employing more diverse sampling methods to broaden the participant pool, ensuring a more representative sample. Additionally, incorporating control groups and randomization into study designs could bolster internal validity. These enhancements would not only strengthen the study's findings but also provide more robust evidence regarding the efficacy of yoga therapy in elderly populations. Overall, this study lays a solid foundation for future research to build upon, ultimately advancing our understanding of the therapeutic potential of yoga in promoting health and well-being among elderly individuals.

## Appendices

Insomnia Severity Index

The Insomnia Severity Index has seven questions. The seven answers are added up to get a total score. When you have your total score, look at the 'Guidelines for Scoring/Interpretation' below to see where your sleep difficulty fits.

For each question, please CIRCLE the number that best describes your answer.

Please rate the CURRENT (i.e. LAST 2 WEEKS) SEVERITY of your insomnia problem(s).

**Table 5:** Insomnia Severity Index questions

Insomnia Problem	None	Mild	Moderate	Severe	Very Severe
1. Difficulty falling asleep	0	1	2	3	4
2. Difficulty staying asleep	0	1	2	3	4
3. Problems waking up too early	0	1	2	3	4

4.  How SATISFIED/DISSATISFIED are you with your CURRENT sleep pattern?

Very Satisfied 0

Satisfied 1

Moderately Satisfied 2

Dissatisfied 3

Very Dissatisfied 4

5.  How NOTICEABLE to others do you think your sleep problem is in terms of impairing the quality of your life?

Not at all Noticeable 0

A Little 1

Somewhat 2

Much 3

Very Much Noticeable 4

6.  How WORRIED/DISTRESSED are you about your current sleep problem?

Not at all Worried 0

A Little 1

Somewhat 2

Much 3

Very Much Worried 4

7.  To what extent do you consider your sleep problem to INTERFERE with your daily functioning (e.g. daytime fatigue, mood, ability to function at work/daily chores, concentration, memory, mood, etc.) CURRENTLY?

Not at all Interfering 0

A Little 1

Somewhat 2

Much 3

Very Much Interfering 4

Guidelines for Scoring/Interpretation:

Add the scores for all seven items (questions 1 + 2 + 3 + 4 + 5 +6 + 7) = your total score Total score categories:

0-7 = No clinically significant insomnia

8-14 = Subthreshold insomnia

15-21 = Clinical insomnia (moderate severity)

22-28 = Clinical insomnia (severe)
